# The Shine-Through Masking Paradigm Is a Potential Endophenotype of Schizophrenia

**DOI:** 10.1371/journal.pone.0014268

**Published:** 2010-12-09

**Authors:** Eka Chkonia, Maya Roinishvili, Natia Makhatadze, Lidia Tsverava, Andrea Stroux, Konrad Neumann, Michael H. Herzog, Andreas Brand

**Affiliations:** 1 Department of Psychiatry, Tbilisi State Medical University, Tbilisi, Georgia; 2 Department of Behaviour and Cognitive Functions, I. Beritashvili Institute of Physiology, Tbilisi, Georgia; 3 Gotsiridze Psycho-Neurological Dispensary, Tbilisi, Georgia; 4 Institute of Biometry and Clinical Epidemiology, CCM, Charité, Berlin, Germany; 5 Laboratory of Psychophysics, Brain Mind Institute, Ecole Polytechnique Fédérale de Lausanne (EPFL), Lausanne, Switzerland; 6 Klinikum Bremen-Ost, Center for Psychiatry and Psychotherapy, Bremen, Germany; Chiba University Center for Forensic Mental Health, Japan

## Abstract

**Background:**

To understand the genetics of schizophrenia, a hunt for so-called intermediate phenotypes or endophenotypes is ongoing. Visual masking has been proposed to be such an endophenotype. However, no systematic study has been conducted yet to prove this claim. Here, we present the first study showing that masking meets the most important criteria for an endophenotype.

**Methodology/Principal Findings:**

We tested 62 schizophrenic patients, 39 non-affected first-degree relatives, and 38 healthy controls in the shine-through masking paradigm and, in addition, in the Continuous Performance Test (CPT) and the Wisconsin Card Sorting Test (WCST). Most importantly, masking performance of relatives was significantly in between the one of patients and controls in the shine-through paradigm. Moreover, deficits were stable throughout one year. Using receiver operating characteristics (ROC) methods, we show that the shine-through paradigm distinguishes with high sensitivity and specificity between schizophrenic patients, first-order relatives and healthy controls.

**Conclusions/Significance:**

The shine-through paradigm is a potential endophenotype.

## Introduction

Schizophrenia is a heterogeneous disease strongly influenced by genetic disposition. A large variety of candidate genes has been identified of which each gene, however, explains only a small proportion of the genetic risk [1]. For this reason, stable markers, so called endophenotypes, are of primary interest. From the original five requirements for an endophenotype [2], one is related to its sensitivity, i.e., an endophenotype should show performance differences between patients and controls and patients with other diseases. Three requirements relate to the genetic underpinnings of the disease of which the most important criterion states that unaffected relatives must show deteriorated performance compared to controls. The fifth criterion requires state independency of the test: performance must not vary across time. Further criteria are practicability and neural explicability [3].

Apart from structural and functional brain measures, several cognitive candidates have been proposed to be endophenotypes for schizophrenia. Other promising candidates rely on visual backward masking where a target is followed by an inter-stimulus interval (ISI) and a mask. The mask impairs performance on the target in both schizophrenic patients and healthy controls. However, masking deficits of patients are much stronger than those of controls. Masking deficits are of particular interest because cognitive deficits may be caused by deficient sensory processing [4]. For decades, masking has been proposed to be a vulnerability marker, supported by longitudinal studies [5], [6], studies with siblings [7]–[10], adolescents [11]–[15], and patients within the schizophrenia spectrum [14], [16].

Whereas there are several masking studies testing *certain* requirements an endophenotype has to meet, none of them is systematic. Many studies compared backward masking performance of patients and controls, very few compared relatives and controls, and none determined masking performance of patients, relatives and healthy controls altogether. One reason might be that most masking techniques determine a wide range of ISIs with only a few trials per ISI which, because of floor and ceiling effects, may not suffice to reliably determine performance differences of *three* populations.

**Figure 1:**
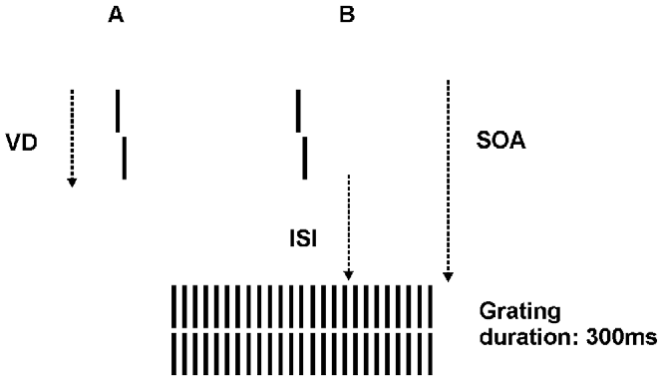
Procedure. (A) For each observer, we determined the individual vernier duration (VD) for which 75% correct responses were reached. (B) In the next step, the vernier was presented with the individual VD of each observer. The vernier offset was fixed at 1.15′. This vernier was followed by a blank screen (ISI) and a grating comprised of 25 aligned verniers, (SOA = VD + ISI).

We have introduced a very sensitive backward masking technique, shine-through, which overcomes floor and ceiling effects by determining the ISI with an adaptive method [17]. In the shine-through effect, a vernier stimulus, i.e. two vertical bars that are slightly offset in the horizontal direction, is presented (figure 1). The task of the observers is to indicate the offset direction of the lower bar compared to the upper bar. After the vernier, a masking grating follows after a variable ISI. With an adaptive method, a critical ISI is determined. This technique yields a very good signal to noise ratio. For example, patients show almost ten times longer ISIs compared to healthy controls [17].

For an endophenotype, it is important that unaffected relatives of patients show performance deficits compared to controls. Hence in the shine-through paradigm, ISIs of relatives should be shorter than ISIs of patients but longer than the ISIs of controls. In addition, performance of an endophenotype should be unaffected by the state of the disease. Hence, masking performance of patients should be constant when tested on different occasions. These predictions were tested here.

We also tested performance in the Continuous Performance Test (CPT) and the Wisconsin Card Sorting Test (WCST) which are frequently used paradigms. Both tests are often proposed to be endophenotypes of schizophrenia [18], [19]. The CPT is sensitive to attention deficits and the WCST is sensitive to deficits of executive functions. We included these tests to compare their power with the power of the shine-through paradigm.

## Methods

### Participants

One hundred schizophrenic patients, 40 first-order relatives and 38 healthy controls joined the study. We excluded 38 patients and 1 relative because vernier durations were too long (see below). Thus, 62 schizophrenic patients, 39 first-order relatives, and 38 healthy controls participated in the main study. Group characteristics are depicted in table 1. All observers had normal or corrected to normal visual acuity as determined with the Freiburg visual acuity test observers had to reach a value of ≥0.8 for at least one eye).

**Table 1:** Demographic data.

	Schizophrenic patients (*N* = 62)		Non-affected first-order relatives (*N* = 39)		Healthy controls (*N* = 38)	
	Mean	*SD*	Mean	*SD*	Mean	*SD*
Female	21/62		26/39		21/38	
Age	35.23	8.05	35.03	15.87	35.39	10.22
Age range	21–52		14–66		20–55	
Education	12.81	2.42	13.15	3.47	15.24	3.03
Illness duration	11.54	8.34				
SANS	10.60	5.56				
SAPS	10.42	3.11				
CPZ	511.3	469.4				
LPS3	17.52	6.09	22.74	5.66	23	6.7
VD	43.2	27.8	27.7	18.98	27.4	16.22

Gender, age (ys), education (ys), duration of illness (ys), SANS (Scale for the Assessment of Negative Symptoms), SAPS (Scale for the Assessment of Positive Symptoms), and vernier duration (VD). There are more males in the schizophrenia group. Controls are better educated than patients and relatives. The VD of schizophrenic patients is nearly twice that of relatives and controls. Indicated are means and standard deviations.

Schizophrenic patients were recruited from the Asatiani psychiatric hospital, the Gotsiridze psycho-neurological dispensary, and the rehabilitation centre. First-order relatives were asked to participate after the patients consented. Healthy controls were recruited from the general population. General exclusion criteria were drug or alcohol abuse, neurological or other somatic mind-altering illnesses. All relatives and controls were free from psychiatric axis I disorders. We did not test for axis II or schizotypic features. To participate, patients and controls had to be older than 18 years and younger than 56 years, relatives 14 to 70 years (to include offspring and parents). The age of the patients ranged from 21 to 52 years, the relatives were between 14 and 66 years old and the controls between 22 and 55 years old (shine-through performance is roughly constant between 15 and 55 years of age [20]). The relatives were 20 siblings, 10 parents, and 9 children (four families contributed with 2 relatives, all other families 1 relative). 37 patients out of 62 did not have any participating relatives. The peer patients of nine of the relatives were excluded because of low visual acuity, long VD, or because they terminated the experiments prematurely.

Ethics approval was obtained from the Georgian National Council on Bioethics. Observers signed informed consent and were informed that they could quit the experiments at any time.

### Diagnosis and psychopathology

The patients were diagnosed using the DSM-IV by means of an interview based on the SCID, information from the staff and the patient records. Psychopathology was assessed by the Scale for the Assessment of Negative (SANS) and positive (SAPS) Symptoms [21], [22] carried out by an experienced senior psychiatrist (EC).

All patients were receiving neuroleptic medication: 41 took biperiden, 8 an additional antidepressant, 7 carbamazepine, and 7 received diazepam. Chlorpromazine equivalents are indicated in table 1.

### Shine-through backward masking test paradigm

The shine-through paradigm is described in detail in [17]. Subjects observed the stimuli from a distance of 3.5 m in a dimly illuminated room. A pixel of the screen comprised about 18” (arcsec) at this distance. The stimuli were white (100 cd/m^2^) on a black background.

The verniers consisted of two vertical bars of 10′ (arc min) length. The lower bar was slightly offset either to the left or right with respect to the upper bar. In each trial, the vernier offset direction was chosen randomly. In a binary task, observers were asked to indicate this offset direction. Errors were indicated by an auditory signal. In the masking condition, a grating followed the vernier. The grating comprised 25 verniers without offset of the same length as the target vernier. The horizontal distance between grating elements was about 3.33′. The vernier and the central element of the grating always appeared in the middle of the screen. Conditions were presented in blocks of 80 trials.

First, we tested unmasked verniers in order to determine the individual vernier duration using a staircase procedure (figure 1; [17]). Observers with vernier durations longer than 100 ms were excluded at this stage to ensure that all observers were rather “good” performers (sensitivity should not be boosted by “bad” performers). 38 out of 100 patients and one relative out of 40 were excluded because of too long vernier durations (VD).

In the masking experiment, we presented the vernier with the individual duration of each observer as determined in the previous step (20 ms was the minimal duration). The vernier offset size was set to 1.15′ for all observers. After the vernier, an ISI followed, i.e. a blank screen, and then the grating for 300 ms (figure 1B). We adaptively assessed the target-mask stimulus-onset-asynchrony (SOA = VD + ISI) which yields a performance level of 75% correct responses. The starting value of the SOA was 200 ms which was either decreased or increased to find the individual threshold. A value of 450 ms was recorded, if observers were unable to reach 75% correct responses for an SOA of 400 ms [23]. This applied to only 2 patients.

### Neuropsychological tests

We administered a computerized version of the Nelson Test [24], a modified WCST with 48 cards, and the degraded Continuous Performance Test CPT-DS [25] with 3 blocks (720 digits, 10% targets, degradation 40%) and a total duration of 12 minutes. Observers had to detect the pair “1-9”. The digits were presented randomly with a rate of one per second with a presentation time of 50 ms. In order to assess general cognitive ability, we administered the LPS3 (Leistungspruefsystem). The LPS3 tests non-verbal cognitive capabilities using abstract shapes [26]. The tests were administered in the following order: shine-through paradigm, WCST, CPT-DS, and LPS3.

### Stability of performance on the shine-through paradigm

To test stability of performance in the shine-through paradigm, we asked patients and controls to participate in a second and a third session about six and twelve months after the first session, respectively (relatives were not tested). We tested only 23 of the patients who participated in the first session (for each participant, we used the same individual VD for the three tests as determined in the first testing). For the third session, 5 patients dropped out because of various reasons (no address, problems of transportation from rural regions).

### Data Analysis

For statistical analysis, we selected one particular meaningful variable for each test. For the shine-through paradigm, we chose the SOA, d' for the CPT-DS [27], and the total number of errors (WCST-Err) for the WCST [28], [29].

To account for possible correlations of SOA, CPT-DS, WCST-Err, and LPS3 between members of a family, the data were subjected to General Estimating Equations (GEE), procedure genmode SAS 9.1. In order to adjust for education, gender and age these variables were included in the model as covariates. The shine-through data (SOA) were log-transformed to obtain normally distributed data (figures and tables were not log-transformed). All other variables were normally distributed.To investigate the diagnostic power of the tests, we computed Receiver Operating Characteristics (ROC) curves and determined the area under the curves (AUC). Differences between the AUCs were tested by non-parametric tests [30]. Sensitivity and specificity were calculated to maximize their sum (sensitivity and specificity relate to comparisons between schizophrenic patients and relatives/controls but not to comparisons with other (psychiatric) diseases). Stability of shine-through performance was analysed by a repeated measures analysis with 3 intra-individual (time points) and one between group factor (patients vs. controls). Additionally, we described the stability by intraclass correlation coefficients in a mixed two-way effects model. The influence of the psychopathological variables (SANS and SAPS) on the SOA was examined by a general linear model.

## Results

Group characteristics are shown in table 1. Controls had higher education levels than relatives (p = 0.006) and patients (p<0.0001). The patient group had a higher proportion of males (41 vs. 21) than the relative group (13 vs. 26) and the control group (17 vs. 21). VDs were almost identical for controls and relatives but higher for the patients (table 1).

**Figure 2:**
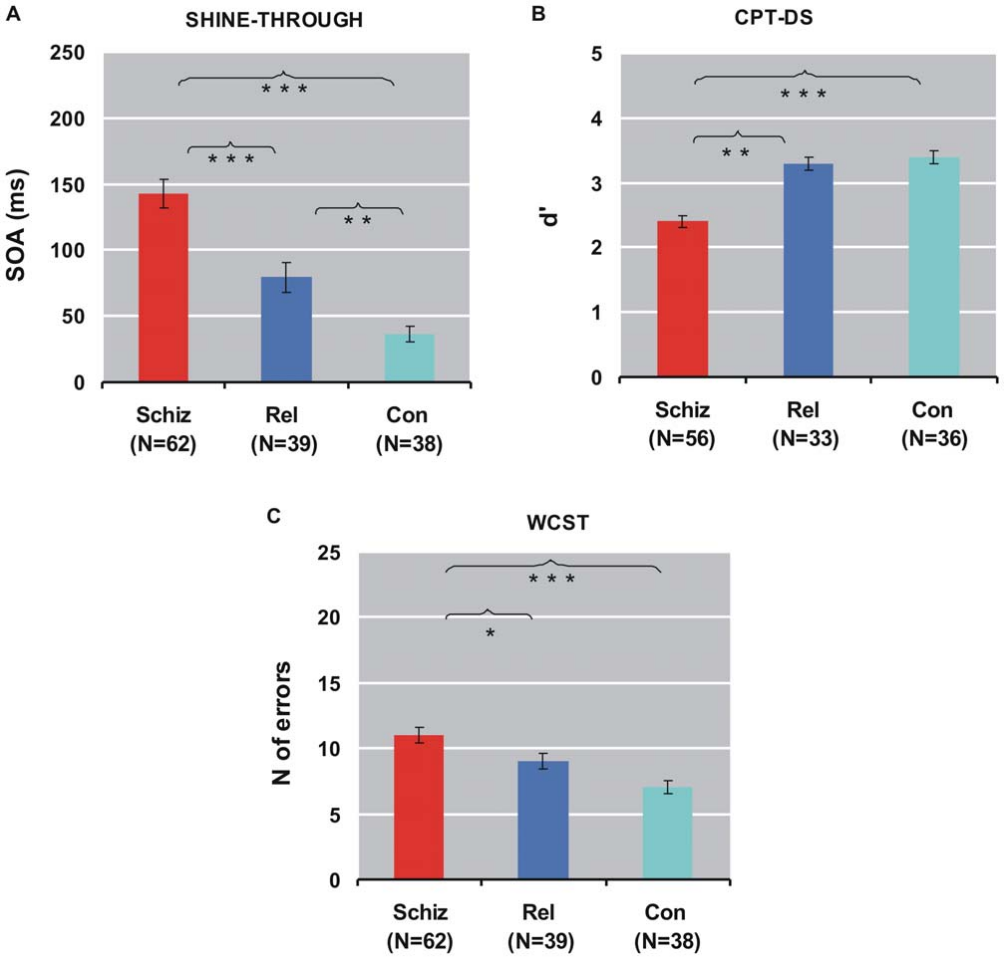
GEE results of shine-through, CPT-DS, and WCST. Dependent variables were SOA (stimulus onset asynchrony), d', and total number of errors, respectively. Bars indicate performance of schizophrenic patients (schiz), non-affected first-order relatives (rel), and healthy controls (con). Error bars indicate standard errors. Schizophrenic patients perform worse than controls in all tests. The difference between relatives and healthy controls is only significant in the shine-through paradigm; * *p*<0.01, ** *p*<0.001, *** *p*<0.0001.

The GEE showed that the group factor was significant in all three tests (*p*<0.0001; figure 2). Schizophrenic patients performed worse than healthy controls (*p*<0.0001, in each test) and relatives (shine-through *p*<0.0001, CPT-DS *p* = 0.0002, WCST errors *p* = 0.009). Relatives performed significantly worse than controls in the shine-through test (*p* = 0.0001) but not in the CPT-DS nor in the WCST (CPT-DS: *p* = 0.55, WCST-Err: 0.16; see figure 2)). Performance in the LPS3 was significantly impaired for patients compared to relatives and controls. There was no significant difference between relatives and controls.

### Power of discrimination

#### Schizophrenic patients vs. healthy controls

**Figure 3:**
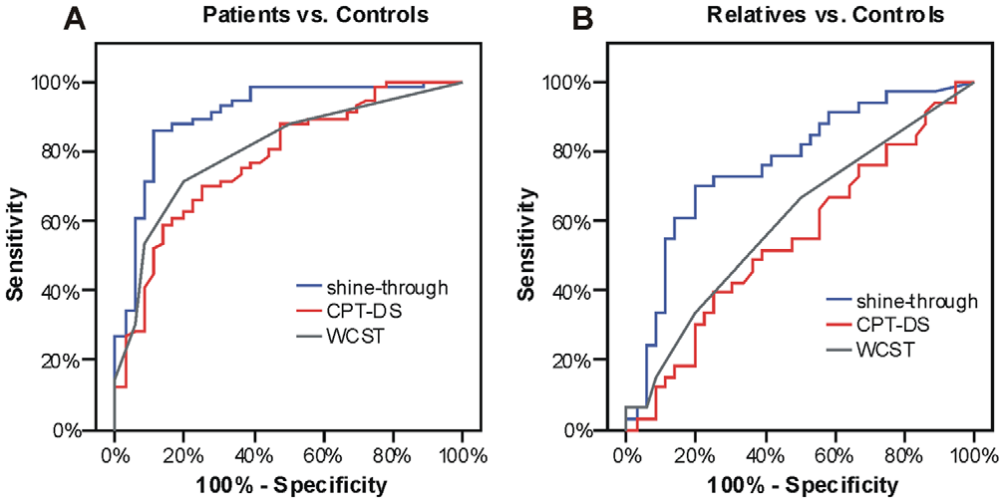
ROC (receiver operating characteristics) curves for the shine-through paradigm, the CPT-DS, and WCST. (A) ROC curves for schizophrenic patients and healthy controls. The x-axis indicates the error of the second kind (100%-specificity). The y-axis indicates sensitivity. The area under the curve (AUC) shows the discriminative power between the two groups. The diagonal from (0,0) to (100,100) with AUC = 0.5 indicates a total lack of discriminative power. AUC values are given in the text. All tests discriminate well between schizophrenic patients and healthy controls with the shine-through paradigm showing the significantly best discrimination. (B) ROC curves for non-affected first-order relatives of schizophrenic patients and healthy controls. All ROC curves are more attenuated than the ones comparing patients and controls (figure 3 A). Again, the shine-through paradigm yields the highest discriminative power.

ROC analysis showed that the shine-through paradigm differentiates better than the other tests between patients and controls (figure 3). The AUC for the shine-through paradigm, the CPT-DS, and the WCST were: 0.916, 0.779, and 0.791, respectively. An AUC of 0.5 indicates the test has no discriminative power. Comparisons of the AUCs by the DeLong test revealed that the shine-through paradigm differentiates significantly better between both groups than the other tests (shine-through vs. CPT-DS: *p* = 0.005; shine-through vs. WCST: *p* = 0.02).

We found a sensitivity of 87% (*p*<0.0005) and a specificity of 89% (*p*<0.0005) for the shine-through paradigm. Sensitivity was 59% for the CPT-DS (*p* = 0.12) and 73% for the WCST (*p*<0.08), specificity 86% for the CPT-DS (*p*<0.002) and 90% for the WCST (*p*<0.0005).

#### Relatives vs. healthy controls

The AUCs show that the shine-through paradigm discriminates well between relatives and controls (0.743). The AUCs related to the other tests were substantially lower (CPT-DS: 0.548; WCST: 0.660). The power of discrimination of the shine-through paradigm was significantly higher than the power of the CPT-DS (*p* = 0.009). There was no significant difference of power between shine-through and the WCST (*p* = 0.35).

For the shine-through test, sensitivity and specificity were 67% (*p* = 0.03) and 82% (*p*<0.0005), respectively. The sensitivity was 39% for the CPT-DS and 46% for the WCST. Specificity was 75% for the CPT-DS (*p* = 0.002) and 82% for the WCST (*p*<0.0005).

#### Stability of shine-through

**Figure 4:**
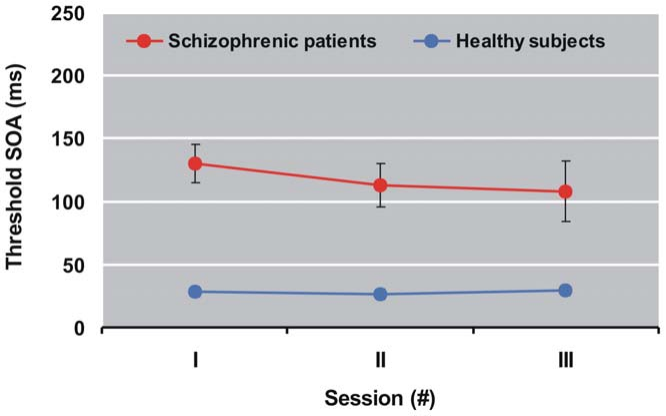
Performance in the shine-through paradigm for 18 schizophrenic patients and 20 controls at three occasions. Session II took place about 6 months after session I and session III about 6 months after session II. Schizophrenic patients show a weak (but statistically non significant) improvement with time. Error bars show standard errors.

18 schizophrenic patients and 20 controls participated in all three sessions (figure 4). A repeated measures analysis showed a decrease of the SOA across time with only a tendency to significance in both groups (*F*[1,71] = 2.97; *p* = 0.06; partial eta-square  = 0.08). Patients performed dramatically worse on all three occasions (*F* [1], [36] = 34.2; *p*<0.0001; partial eta-square  = 0.49). There was no significant interaction of group and time. Intraclass correlations were 0.67 (patients) and 0.74 (controls) between the first and the third test session.

Psychopathological measures in the schizophrenic patients changed moderately between sessions. SAPS decreased from 9.8 to 8.6 whereas SANS increased from 8.4 to 11.3. A mixed linear model revealed no significant influence of SANS and SAPS on performance in the shine-through paradigm.

## Discussion

For decades, visual masking is suggested to be a vulnerability marker [4]. Until now, there was no systematic masking study where the major criteria of an endophenotype within a *single* paradigm were tested. Here, we showed the shine-through paradigm is a potential endophenotype because it meets the major criteria proposed by Gottesman and Gould [2] and the practicability and explicability criteria proposed by Turetsky et al. [3].

### The shine-through paradigm and the endophenotype concept

1. Association with the disease [2]. Our results show that the shine-through paradigm is associated with schizophrenia revealing a remarkable sensitivity of 87% and specificity of 89%. Also the effect size of the shine-through test, as compared between patients and controls, shows a large value of 1.96 (table 2). Furthermore, we excluded patients with VDs longer than 100 ms. Hence, our patients are rather good performers. Including bad performers might even have increased sensitivity and specificity. Our results are in good agreement with most masking studies which have consistently shown that performance of schizophrenic patients is deteriorated compared to controls. However, we cannot compare the discriminative power of the shine-through paradigm with that of other masking paradigms because in these studies sensitivity and specificity were never computed.

**Table 2:** Effect sizes (ES) and confidence intervals (CI) of the comparisons between relatives and patients, relatives and controls, and patients and controls.

	ES	CI 95%		ES	CI 95%	ES	C I 95%	Relative effect size (partial eta-square)
	relatives vs. patients			relatives vs. controls		patients vs. controls		
Shine-through (log-transformed)	0.752		0.408–1.095	0.761	0.420–1.101	1.513	1.225–1.801	0.420
CPT	0.968		0.562–1.374	0.120	−0.277–0.518	1.088	0.667–1.510	0.234
WCST (Errors)	2.287		0.822–3.751	1.258	−0.236–2.751	3.545	2.110–4.980	0.118

The SOA of the shine-through test is log-transformed. ES =  Mean difference adjusted by age, gender, and education (GEE). Partial eta-squared values are estimated by the general linear model.

Unmasked VDs were prolonged in the schizophrenic patients compared to controls. We compensated for this effect by providing individual VDs in the masking conditions as most studies on masking do. It has been proposed that vernier *acuity* itself is an endophenotype [31], [32]. In our study, unmasked vernier *durations* were not significantly longer in relatives of patients compared to controls (however, [31], [32] used much longer VDs).

2. Relatives [2]. Non-affected first-order relatives are significantly impaired in the shine-through test compared to controls. Thus, the shine-through test discriminates well between patients and controls *and* between relatives and healthy controls. Also the effect size between relatives and controls was large for the shine-through test (0.99).

The relatives group had a wider age range and a lower education level than controls. However, GEE with age and education as covariates still yielded significant differences between relatives and controls (figure 2). Moreover, education did not correlate with shine-through performance; global cognitive ability (LPS3) showed no significant difference between relatives and controls.

3. Heritability [2]. Our study was not designed to investigate the heritability of the shine-through test. One recent study of heritability of backward masking, different from the shine-through paradigm, has shown moderate familial correlations [33].

4. State independency. Two studies determined stable masking performance for a period of more than 1.5 years [5], [6]. Also in our study masking performance was roughly stable throughout one year. Intraclass correlation coefficients show a high stability across time (however, patients showed only a weak clinical recovery).

5. Practicability [3]. The shine-through paradigm is easily *applicable* because it can be applied with two blocks of measurements each lasting less than 5 min. VD estimation takes another 10–20 min.

6. In addition, the shine-through paradigm offers insights into the mechanisms of deficient visual information processing of schizophrenic patients. For example, schizophrenic patients are not simply slower in processing visual stimuli. It seems that patients have problems suppressing mask information [34]–[36]. The shine-through effect can be understood in terms of low level neural interactions as shown by computer simulations [37], [38].

### Methodological considerations

Visual masking in general is a very promising candidate for an endophenotype as many previous studies have shown. In most of these studies, a certain number of ISIs are pre-selected and percent correct or the (bias prone) hit rate is determined per ISI. To keep the experiment feasible, only a few trials per ISI are presented. This often yields a weak signal to noise ratio with one of the populations performing either in the floor or ceiling region. Therefore, it is unlikely that performance differences of three populations with very different performance levels can reliably be determined. However, this is important for an endophenotype and for genetic analysis. For this reason, we propose that using an adaptive method without predefined ISIs is a more efficient strategy because the adaptive method “chooses” the ISIs which are most informative for each participant individually.

We also tested performance in the CPT and the WCST which are frequently used tests often proposed to be endophenotypes of schizophrenia [18], [19]. We included these test to compare their power with the power of the shine-through paradigm. We found that the shine-through paradigm has a higher sensitivity and specificity than both the CPT and WCST for both the comparison between controls and patients and relatives of patients and controls.

### Limitations

First, all patients received medication. However, backward masking does usually not deteriorate with drug application [39]. Second, controls were better educated than patients and relatives, the age distribution of the relatives was not well matched with that of the controls, and the gender distribution varied between the groups (e.g. the patient group containing more males than the relatives group). However, GEE analyses with gender, education, and age as covariates still yielded significant differences between relatives and controls for the shine-through test. A third limitation of the study is the rather small sample size of first-order relatives.

### Summary

The shine-through paradigm is a potential endophenotype for schizophrenia having both a high sensitivity and specificity for detecting performance differences between patients and controls and between relatives and controls. Future research has to probe whether masking deficits are associated with genetic variants in the patients. The genetic variants and related neurophysiological mechanisms underlying the masking deficits may be different from those of other endophenotypes, such as the WCST. Thus, different endophenotypes may lead to the characterization of subgroups of schizophrenia.

## References

[1] Sanders AR, Duan J, Levinson DF, Shi J, He D (2008). No significant association of 14 candidate genes with schizophrenia in a large European ancestry sample: implications for psychiatric genetics.. Am J Psychiatry.

[2] Gottesman II, Gould TD (2003). The endophenotype concept in psychiatry: Etymology and strategic intentions.. Am J Psychiatry.

[3] Turetsky BI, Calkins ME, Light GA, Olincy A, Radant AD (2007). Neurophysiological Endophenotypes of Schizophrenia: The Viability of Selected Candidate Measures.. Schiz Bull.

[4] Braff DL, Saccuzzo DP (1981). Information processing dysfunction in paranoid schizophrenia: a two-factor deficit.. Am J Psych.

[5] Lee J, Nuechterlein KH, Subotnik KL, Sugar CA, Ventura J (2008). Stability of visual masking performance in recent-onset schizophrenia: An 18-month longitudinal study.. Schiz Res e-pub.

[6] Rund BR, Landrø NI, Orbeck AL (1993). Stability in backward masking performance in schizophrenics, affectively disturbed patients, and normal subjects.. J Nerv Ment Dis.

[7] Bedwell JS, Brown JM, Miller LS (2003). The magnocellular visual system and schizophrenia: what can the color red tell us?. Schiz Res.

[8] Green MF, Nuechterlein KH, Breitmeyer B (1997). Backward masking performance in unaffected siblings of schizophrenic patients. Evidence for a vulnerability indicator. Arch Gen Psychiatry 54: 465-472.. Erratum in: Arch Gen Psychiatry 1997.

[9] Green MF, Nuechterlein KH, Breitmeyer B, Mintz J (2006). Forward and backward visual masking in unaffected siblings of schizophrenic patients.. Biol Psychiatry.

[10] Keri S, Kelemen O, Benedek G, Janka Z (2001). Different trait markers for schizophrenia and bipolar disorder: a neurocognitive approach.. Psychol Med.

[11] Holzer L, Jaugey L, Chinet L, Herzog MH (2009). Deteriorated visual backward masking in the shine-through effect in adolescents with psychosis.. J Clin Exp Neuropsychol.

[12] Lieb K, Denz E, Hess R, Schüttler R, Kornhuber HH (1996). Preattentive information processing as measured by backward masking and text on detection tasks in adolescents at high genetic risk for schizophrenia.. Schiz Res.

[13] Rund BR, Øie M, Sundet K (1996). Backward-masking deficit in adolescents with schizophrenic disorders or attention deficit hyperactivity disorder.. Am J Psych.

[14] Saccuzzo DP, Schubert DL (1981). Backward masking as a measure of slow processing in schizophrenia spectrum disorders.. J Abnorm Psychol.

[15] Ueland T, Øie M, Landrø NI, Rund BR (2004). Cognitive functioning in adolescents with schizophrenia spectrum disorders.. Psych Res.

[16] Merritt RD, Balogh DW (1990). Backward masking as a function of spatial frequency. A comparison of MMPI-identified schizotypics and control subjects.. J Nerv Ment Dis.

[17] Herzog MH, Kopmann S, Brand A (2004). Intact figure-ground-segmentation in schizophrenic patients.. Psych Res.

[18] Gur RE, Calkins ME, Gur RC, Horan WP, Nuechterlein KH, Seidman LJ, Stone WS (2007). The Consortium on the Genetics of Schizophrenia: Neurocognitive Endophenotypes.. Schiz Bull.

[19] Birkett P, Sigmundsson T, Sharma T, Toulopoulou T, Griffiths TD, Reveley A, Murray R (2008). Executive function and genetic predisposition to schizophrenia _the Maudsley family study.. Am J Med Genet B Neuropsychiatr.

[20] Parkosadze K, Kunchulia M, Roinishvili M, Herzog MH (2009). Age-related differences in temporal processing..

[21] Andreasen NC (1983). Scale for the Assessment of Negative Symptoms (SANS)..

[22] Andreasen NC (1984). Scale for the Assessment of Positive Symptoms (SAPS)..

[23] Herzog MH, Fahle M, Koch C (2001). Spatial aspects of object formation revealed by a new illusion, shine-through.. Vis Res.

[24] Nelson H (1976). A modified card sorting response sensitive to frontal lobe defects.. Cortex.

[25] Rosvold H, Mirsky A, Sarason I, Bransome E, Beck L (1956). A continuous performance test of brain damage.. J Consul Clin Psychol.

[26] Horn W (1983). Leistungspruefsystem LPS Handanweisung fuer die Durchfuehrung, Auswertung und Interpretation.. erweiterte und verbesserte Auflage.

[27] MacMillan NA, Creelman CD (1991). *Detection theory: A user's guide*..

[28] Snitz BE, Macdonald AW, Carter CS (2006). Cognitive deficits in unaffected first-degree relatives of schizophrenia patients: a meta-analytic review of putative endophenotypes.. Schiz Bull.

[29] Szöke A, Schürhoff F, Mathieu F, Meary A, Ionescu S (2005). Tests of executive functions in first-degree relatives of schizophrenic patients: a meta-analysis.. Psychol Med.

[30] DeLong ER, DeLong DM (1988). Clarke-Pearson DL comparing the areas under two or more correlated receiver operating characteristic curves: a nonparametric approach.. Biometrics.

[31] Kéri S, Kelemen O, Benedek G, Janka Z (2004). Vernier threshold in patients with schizophrenia and in their unaffected siblings.. Neuropsycholog*y*.

[32] Keri S, Kelemen O, Benedek G, Janka Z (2005). Lateral interactions in the visual cortex of patients with schizophrenia and bipolar disorder.. Psychol Med.

[33] Aukes MF, Alizadeh BZ, Sitskoorn MM, Selten J, Sinke RJ (2008). Finding Suitable Phenotypes for Genetic Studies of Schizophrenia: Heritability and Segregation Analysis.. Biol Psychol.

[34] Brand A, Kopmann S, Herzog MH (2004). Intact feature fusion in schizophrenic patients.. Eur Arch Psychiatry Clin Neurosci.

[35] Roinishvili M, Chkonia E, Brand A, Herzog MH (2008). Contextual suppression and protection in schizophrenic patients.. Eur Arch Psychiatry Clinl Neurosci.

[36] Schutze C, Bongard I, Marbach S, Brand A, Herzog MH (2007). Collinear contextual suppression in schizophrenic patients.. Psych.

[37] Hermens F, Luksys G, Gerstner W, Herzog MH, Ernst U (2008). Modeling spatial and temporal aspects of visual backward masking.. Psychol Rev.

[38] Herzog MH, Ernst U, Etzold A, Eurich C (2003). Local interactions in neural networks explain global effects in gestalt processing and masking.. Neural Comput.

[39] Brody D, Saccuzzo DP, Braff DL (1980). Information processing for masked and unmasked stimuli in schizophrenia and old age.. J Abnorm Psychol.

